# Ozone oxidation of antidepressants in wastewater –Treatment evaluation and characterization of new by-products by LC-QToFMS

**DOI:** 10.1186/1752-153X-7-15

**Published:** 2013-01-25

**Authors:** André Lajeunesse, Mireille Blais, Benoît Barbeau, Sébastien Sauvé, Christian Gagnon

**Affiliations:** 1 Environment Canada, Wastewater and Effluents Section, Water Science and Technology Directorate, 105 McGill Street, Montreal, Quebec, H2Y 2E7, Canada; 2 École Polytechnique de Montréal, Department of Civil, Geological and Mining Engineering, P.O. Box 6079, Succursale Centre-ville, Montreal, Quebec, H3C 3A7, Canada; 3 Department of Chemistry, Université de Montréal, P.O. Box 6128, Succursale Centre-ville, Montreal, Quebec, H3C 3J7, Canada

**Keywords:** Antidepressants, Ozone, LC-MS/MS, Sewage treatment plants, Biosolids, Side-products

## Abstract

**Background:**

The fate of 14 antidepressants along with their respective *N*-desmethyl metabolites and the anticonvulsive drug carbamazepine was examined in a primary sewage treatment plant (STP) and following advanced treatments with ozone (O_3_). The concentrations of each pharmaceutical compound were determined in raw sewage, effluent and sewage sludge samples by LC-MS/MS analysis. The occurrence of antidepressant by-products formed in treated effluent after ozonation was also investigated.

**Results:**

Current primary treatments using physical and chemical processes removed little of the compounds (mean removal efficiency: 19%). Experimental sorption coefficients (K_d_) of each studied compounds were also calculated. Sorption of venlafaxine, desmethylvenlafaxine, and carbamazepine on sludge was assumed to be negligible (log K_d_ ≤ 2), but higher sorption behavior can be expected for sertraline (log K_d_ ≥ 4). Ozonation treatment with O_3_ (5 mg/L) led to a satisfactory mean removal efficiency of 88% of the compounds. Screening of the final ozone-treated effluent samples by high resolution-mass spectrometry (LC-QqToFMS) did confirm the presence of related *N*-oxide by-products.

**Conclusion:**

Effluent ozonation led to higher mean removal efficiencies than current primary treatment, and therefore represented a promising strategy for the elimination of antidepressants in urban wastewaters. However, the use of O_3_ produced by-products with unknown toxicity.

## Background

Urban wastewaters are one of the major sources of pharmaceutically-active compounds (PhACs) into aquatic environments [1,2]. The elimination of many pharmaceuticals in sewage treatment plants (STPs) being often incomplete [3-5], effluents from STPs thus contribute to a significant load of pharmaceutical residues in the receiving waters [6]. Little is however known on the potential release of transformation by-products following advanced wastewater treatments.

Among the most prescribed PhACs throughout the world are the psychiatric drugs that include the antidepressants and the antiepileptic drug carbamazepine (CAR) frequently used for treating schizophrenia and bipolar disorder [7,8]. The persistent drug CAR largely sold in Canada is currently prescribed in combination to antidepressants all over the world during therapy. Therefore, a monitoring of CAR is also required to better assess its environmental fate in different matrices. Toxicity studies of these neuroactive compounds provided evidence for biological effects on aquatic organisms [9-13]. Although the occurrence of antidepressants in sewage effluents [6,14-17] and wastewater sludge [18-20] has been demonstrated, the fate of these substances following different treatments in STPs has not been extensively documented. A previous study indicated that a primary treatment process has limited capability to remove and/or degrade antidepressants residues in wastewater [15]. Further results obtained for STPs operating different biological processes (e.g. secondary treatment with activated sludge) revealed moderate potential (mean removal efficiency ≤ 30%) to degrade antidepressants from wastewater [20]. Therefore, alternative treatment technologies may have to be implemented or combined to achieve high removal of compounds in STPs [21]. As such, experimental evidence reported elsewhere clearly demonstrates that existing limitations in primary and secondary processes can be overcome with more advanced treatment strategies including chemical oxidation with ozone or the use of high pressure membrane technologies [22-24].

While conventional activated sludge treatments were shown to degrade pharmaceuticals to varying extent [25], ozone (O_3_) treatments showed promising results in terms of removal efficiencies as an efficient oxidizer to remove endocrine disruptors compounds and pharmaceuticals products in wastewater [26,27]. Generally, O_3_ reacts with organic molecules through either the direct reaction with molecular O_3_ (via 1–3 dipolar cyclo addition reaction on unsaturated bonds, and electrophilic reaction on aromatics having electron donor groups e.g. OH, NH_2_) or by decomposition through the formation of chain intermediate free radicals, including the hydroxyl radical OH· (less selective reaction on saturated aliphatic molecules) [26,28]. The stability of dissolved ozone is readily affected by pH, ultraviolet (UV) light, ozone concentration, and the concentration of radical scavengers such carbonate – bicarbonate species, the dissolved organic carbon and humic acids [28,29]. Except for few experiments completed with fluoxetine (FLU), the number of studies dedicated to the elimination of antidepressants by oxidation processes (e.g. TiO_2_ membrane reactor, O_3_ with UV activation, O_3_ with H_2_O_2_) has been rather limited [22-24]. Since molecular O_3_ is a selective electrophile that reacts quickly with amine and double bounds moieties [26], ozonation should be efficient to degrade antidepressants mostly constituted of secondary or tertiary amine and conjugated rings. However, as reported for β-Lactam antibacterial agents (e.g. penicillin G, cephalexin) spiked in wastewater, O_3_ reaction leads to the formation of biologically active sulfoxides analogues [30]. For antidepressants, no study on the transformation products following an O_3_ treatment in wastewater is currently available. As yet, no data is reported neither on by-products toxicity. Nevertheless, formation of *N*-oxide, amide, aldehyde, and carboxylic acid by-products is expected after ozonation of secondary and tertiary amine compounds in aqueous solutions [31,32].

In the present work, the effectiveness of ozone treatments in terms of removal efficiency is tested at three different concentrations for the oxidation of 14 antidepressants along with their direct *N*-desmethyl metabolites and the anticonvulsive drug carbamazepine during ozonation of a primary-treated effluent. The goal of the study was also to investigate the occurrence of antidepressant by-products formed in treated effluent after ozonation.

## Experimental

### Chemicals and materials

**Figure 1:**
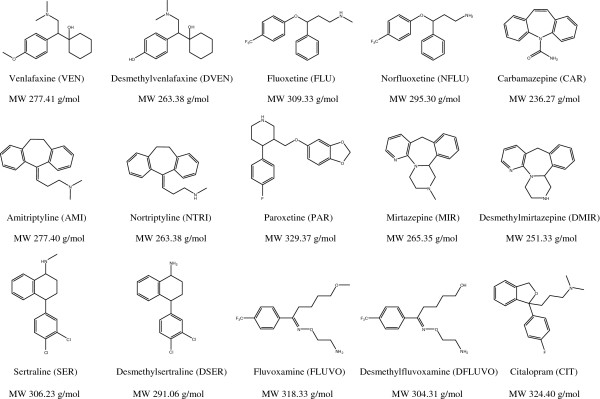
Chemical structures of the studied compounds.

All certified standards were > 98% purity grade. Fluoxetine (FLU), norfluoxetine (NFLU), paroxetine (PAR), sertraline (SER), (*S*)-citalopram (CIT), fluvoxamine (FLUVO), desmethylfluvoxamine (DFLUVO), mirtazapine (MIR), and desmethylmirtazepine (DMIR) were provided by Toronto Research Chemicals Inc. (North York, Ontario, Canada). Desmethylsertraline (DSER), venlafaxine (VEN), *O*-desmethylvenlafaxine (DVEN), and the surrogate standard bupropion-*d*_9_ were obtained from Nanjing Jinglong PharmaTech (Nanjing, China). Amitriptyline (AMI), nortriptyline (NTRI), carbamazepine (CAR), and surrogate standard 10,11-dihydrocarbamazepine were purchased from Sigma-Aldrich Co. (St. Louis, Missouri, USA), while internal standard cis-tramadol^13^C-*d*_3_ was purchased from Cerilliant Corp. (Round Rock, Texas, USA). The high-performance liquid chromatography–grade solvents (methanol and acetonitrile) and ammonium hydroxide were provided by Caledon Laboratories Ltd. (Georgetown, Ontario, Canada). Reagent-grade hydrochloric acid, acetic acid, ammonium bicarbonate, and ACS grade ethyl acetate were provided by American Chemicals Ltd. (Montreal, Quebec, Canada). Solid-phase extraction (SPE) cartridges of 6 mL, 200 mg Strata™ X-C were purchased from Phenomenex (Torrance, California, USA). Stock solutions of 100 mg/L of each substance were prepared in methanol and stored at 4°C in amber glass bottles that were previously washed with methanol. The chemical structures of the selected compounds are provided in Figure 1.

### Instrumentation

#### Liquid chromatography (LC)

Liquid chromatography (LC) was performed using an Agilent 1200 Series LC system equipped with binary pumps, degasser, and a thermostated autosampler maintained at 4°C. The antidepressants were separated on a Kinetex® XB-C18 column (100 mm × 2.10 mm, 1.7 μm) using a binary gradient made of (A) ammonium bicarbonate (5 mM) pH 7.8, and (B) acetonitrile at a flow rate of 400 μL/min. The volume of injection was 15 μL for influent, effluent, and sludge extracts. The gradient used was (%B): 0 min (10%), 6 min (80%), 10 min (80%), 12 min (90%), 14 min (10%), and 16 min (10%). An equilibration time of 4 min was used resulting in a total run time of 20 min. The column temperature was maintained at 40°C.

#### Tandem-mass spectrometry (QqQMS, QqToFMS)

For quantitative analysis, the LC system was coupled to a 6410 triple quadrupole mass spectrometer (QqQMS) manufactured by Agilent Technologies (Santa Clara, CA, USA) equipped with an electrospray ionization (ESI) source. The capillary was maintained at 4000 V, and the cone voltage was optimized for each compound in the positive-ion mode (ESI+). Additional detector parameters were held constant for all antidepressants: gas temperature 325°C; gas flow 10 L/min; nebulizer 35 psi and dwell time 50 ms. For qualitative by-products analysis, a 6530 quadrupole time-of-flight mass spectrometer (QqToFMS) also manufactured by Agilent Technologies, was utilized. The QqToFMS was equipped with a thermal gradient focusing ESI source (Jet Stream technology). Source parameters consisted of the following: gas temperature 325°C; sheath gas temperature 350°C; sheath gas flow 11 L/min; drying gas flow 5 L/min; nebulizer 35 psig, fragmentor 100 V and capillary voltage 4000 V. The QqToFMS was operated in the 4 GHz High Resolution mode with a low mass range (1700 *m/z*). Purine (121.050873 *m/z*) and Hexakis (922.009798 *m/z*) were used as internal reference masses to improve mass accuracy. Initial tests were performed on treated effluent extracts in high resolution tandem MS mode using a mass range of *m/z* 100–400 (specific collision energy: 0 V) at a rate of 5 spectra/s to screen the exact [M+H]^+^ masses of the precursor ions. Identified compounds were then fragmented with different specific collision energies varying between 0 and 10 V. For both detection systems, the MassHunter software from Agilent Technologies was used for data acquisition and processing. Optimized parameters for QqQMS are listed in a table (Additional file 1).

### Sample location and collection

#### Sample location

All samples were collected onsite at the sewage treatment plant (STP) of the city of Repentigny (30 km North-East of Montreal, Qc, Canada) in amber glass bottles previously washed with methanol during an ozonation pilot-study performed in June 2011. The Repentigny STP typically treats 25 000 m^3^ of raw sewage daily for a population of approximately 60 000 persons. Wastewater is primary-treated using both physical and chemical treatments (e.g. flocculation of suspended matters with alum and/or FeCl_3_). For the purpose of this study, treated wastewater was further experimentally ozone-oxidized on site. Main characteristics of the Repentigny STP are reported in Table 1. Ozonation of the effluent consisted of an ozone (O_3_) generator (Ozone Solution, Model: TG10–Ozone Solution) fed with ultra-pure oxygen (99.9999%). Gaseous ozone was bubbled in a ceramic diffuser located inside a vertical column (6.3 m, 5.08 internal diameter) where both gas transfer and contact time occurred simultaneously. The water flow was maintained at 1.2 L/min_,_ while the O_3_ flow rate injection was kept around 75 to 110 N mL/min (head pressure: 10 psi). Contact time of O_3_ with treated effluent was 10 min. Ozone transfer was monitored by measuring off-gas ozone concentrations using the standard KI procedure [33]. Applied ozone dosages were then corrected for ozone transfer efficiency which varied from 75 to 80%. Total and residual dissolved O_3_ concentrations were determined following the standard indigo trisulfonate colorimetric method [34]. 

**Table 1:** Main water characteristics of the Repentigny sewage treatment plant

**Wastewater**	**Temperature (°C)**	**pH**	**Alkalinity (mg/L) CaCO**_**3**_	**TSS (mg/L)**	**BOD**_**5**_**(mg/L)**	**COD (mg/L)**
Raw sewage (Influent)	17	7.3	189	146	136	227
Effluent	–	7.2	165	12	36	59

TSS: Total Suspended Solids, BOD_5_: Biochemical Oxygen Demand, COD: Chemical Oxygen Demand.

#### Sample collection

Typically, water samples of influent (raw sewage), primary-treated effluent, and ozone treated effluent were collected between 10:00 and 14:00 in polyethylene containers and stored on ice. Samples of wet primary sewage sludge (biosolids) were also collected on the same days and immediately stored on ice in polyethylene bottles. In the laboratory, approximately 10 g of wet biosolid material was filtered with a 0.7 μm glass fiber filter to get a dewatered sludge sample that was frozen, freeze-dried, and stored at −80°C until use. All samples were extracted and analyzed within 48 h after their collection.

### Sample extraction

#### Sewage samples

Extraction method for raw sewage and effluent samples to be analyzed for various classes of antidepressants was done as previously described [15]. The decision to incorporate the neutral drug carbamazepine (CAR) amongst the basic antidepressants forced us to modify the protocol by replacing the strong cation exchange cartridge by a mixed-mode cartridge for sample purification (Strata X-C, Phenomenex) [20].

The validated extraction protocol used here was similar to that described in Lajeunesse et al. [20]. Each 250 mL of filtered sewage sample were spiked with 100 μL of a surrogate standard solution prepared in methanol (bupropion-*d*_9_ / 10,11-dihydrocarbamazepine, 2.5 mg/L) and addition of 2.5 mL of methanol before lowering the pH to around 3 with 100 μL of phosphoric acid (85%). The mixed-mode solid phase extraction (SPE) cartridges were conditioned with 4 mL of methanol followed by at least 8 mL of Milli-Q water. SPE was performed with a VAC ELUT SPS24 manifold (Varian) at flow rates ~10–15 mL/min. After extraction, all cartridges were washed with 2 mL of HCl (0.1 M). The CAR molecules were eluted first with 2 × 2 mL of ethyl acetate prior the evaporation of the solvent in the tubes to dryness under a gentle stream of nitrogen. Meanwhile, all SPE cartridges were washed with 2 mL of methanol. The antidepressants retained onto the sorbent were then eluted with 2 × 2 mL of a solution of 5% (v/v) NH_4_OH in methanol. The combined fractions (e.g. CAR and antidepressants) were mixed with 100 μL of a solution of cis-tramadol^13^*d*_3_ in methanol (5 mg/L) as the internal standard and the solvent in tubes was evaporated to dryness with nitrogen. The dried extracts were reconstituted with 0.50 mL of the mobile phase solution of ammonium bicarbonate (5 mM) pH 7.8 – acetonitrile (1:1 v/v) in injection vials and later injected in LC-QqQMS or LC-QqToFMS for analysis.

#### Sewage sludge samples

The simultaneous extraction of CAR and antidepressants in biosolid samples was completed using the validated protocol reported in Lajeunesse et al. [20]. Briefly, 0.200 g of freeze-dried sludge is transferred to a 16 × 150 mm borosilicate glass screw-top conical tube before adding 8 mL of a solution composed of methanol / 0.1 M acetic acid buffer solution pH 4.0 (1:1 v/v). Each tube were spiked with 100 μL of a surrogate standard solution prepared in methanol (bupropion-*d*_9_ / 10,11-dihydrocarbamazepine, 2.5 mg/L). Samples were then shaken vigorously and mixed on a rotary extractor (Caframo REAX) for 15 min. After extraction, tubes were placed in a sonication bath for 15 min before adding 4 mL of Milli-Q water to each tube. Tubes were then centrifuged (320 x g) at room temperature for 5 min. Following the SPE protocol described previously for aqueous sewage samples, supernatants were transferred directly on mixed-mode cartridges. The final extracts were reconstituted in 0.5 mL of the mobile phase solution of ammonium bicarbonate (5 mM) pH 7.8 – acetonitrile (1:1 v/v), filtered with a PTFE 0.45 μm filter, and then injected in LC-QqQMS system for analysis.

## Results and discussion

### Antidepressants in raw sewage and primary-treated effluent

**Figure 2:**
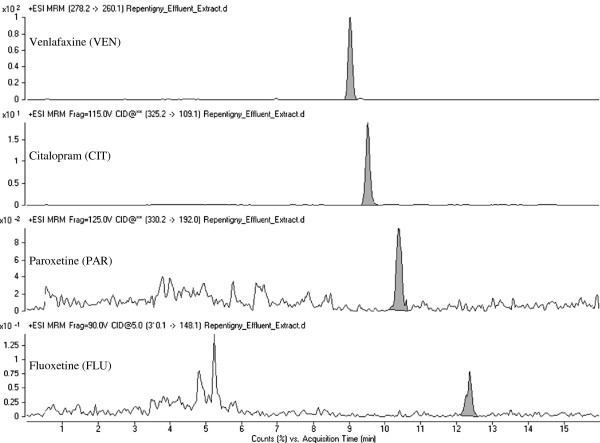
Representative LC-QqQMS chromatograms of selected antidepressants detected in primary-treated effluent sample extract.

Out of the 15 compounds investigated, 13 were detected in raw sewage samples and only the antidepressant FLUVO and its direct metabolite DFLUVO were not detected. Compound concentrations ranged from 6.5 ng/L (NFLU) to 4185 ng/L (DVEN) (Table 2). A typical chromatogram of the detected antidepressants VEN, CIT, PAR, and FLU in a primary-treated effluent extract is depicted in Figure 2. Overall, moderate to poor removal efficiencies were obtained for most antidepressants (mean removal efficiency of 19%). Results showed that current enhanced primary treatment using physical and chemical processes removed little of the studied compounds (Table 2). The substances with lowest removal efficiencies were CAR (4.4%), along with the antidepressant metabolites NTRI (6.8%) and NFLU (7.1%). Similar low removal rates were previously reported for antidepressants [15] and CAR [35] in primary-treated effluents. Despite a noteworthy reduction of total suspended solids – TSS (Table 1), the weak removal obtained for this primary treatment strongly suggests that a mechanism other than chemical adsorption would be required to effectively remove antidepressants from urban wastewater. 

**Table 2:** Mean concentrations of studied compounds extracted in wastewater (raw sewage, effluent) and biosolid samples from the Repentigny STP

**Compounds**	**Wastewaters (n = 2)**			**Biosolids (n = 2)**		
	Raw sewage (ng/L)	Effluent (ng/L)	Removal Eff. (%)	Sludge (ng/g)	K_d_ (L/kg)	log K_d_
CIT	207 ± 12	148 ± 16	29	172 ± 38	1.2 × 10^3^	3.1
SER	13 ± 1	9.4 ± 0.1	28	43 ± 5	4.6 × 10^3^	3.7
DSER	23 ± 1	19 ± 3	17	31 ± 6	1.6 × 10^3^	3.2
AMI	223 ± 21	195 ± 11	13	58 ± 22	2.9 × 10^2^	2.5
NTRI	21 ± 3	19 ± 4	6.8	9.0 ± 1.1	4.7 × 10^2^	2.7
VEN	4061 ± 153	3144 ± 107	23	227 ± 49	7.2 × 10^1^	1.9
DVEN	4185 ± 133	3448 ± 279	18	73 ± 2	2.1 × 10^1^	1.3
CAR	747 ± 14	714 ± 13	4.4	26 ± 12	3.6 × 10^1^	1.6
FLU	11 ± 1	9.5 ± 0.6	16	15 ± 1	1.6 × 10^3^	3.2
NFLU	7.0 ± 0.4	6.5 ± 0.2	7.1	3.8 ± 0.6	5.8 × 10^2^	2.8
PAR	15 ± 1	13 ± 4	9.0	5.6 ± 3.6	4.2 × 10^2^	2.6
MIR	171 ± 20	109 ± 3	36	27 ± 6	2.5 × 10^2^	2.4
DMIR	41 ± 1	25 ± 1	38	13 ± 1	5.4 × 10^2^	2.7

### Antidepressants in sewage sludge

Primary sludge samples consistently displayed quantifiable amounts of the studied compounds (excepted FLUVO and DFLUVO) (Table 2). Highest mean concentrations in biosolid samples were found for VEN (227 ng/g), CIT (172 ng/g), DVEN (70 ng/g), AMI (58 ng/g), and SER (43 ng/g). Our results are consistent with the mean concentrations for the antidepressants FLU (123 ng/g) and PAR (41 ng/g) reported by Radjenović et al. [18] in primary sludge samples. Interestingly, among reported concentrations, less antidepressant metabolites were detected in sewage sludge samples for *N*-desmethyl metabolites in comparison to their respective parent molecules. These findings suggest that more polar compounds have a lower affinity for the solid phase of sewage sludge and hence have limited removal efficiencies.

**Figure 3:**
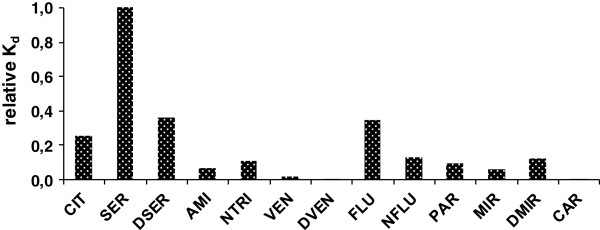
**Relative K**_**d **_**values of the studied compounds.**

In order to describe the fate and behavior of antidepressants in primary STP, specific partitioning coefficient (K_d_) values for antidepressants and metabolites to sewage sludge were estimated. The K_d_ coefficients were calculated using the ratio [Sludge] / [Effluent]; where [Sludge] is the concentration of antidepressants in sewage sludge (ng/kg) and [Effluent] is the concentrations of antidepressants in final effluent (ng/L) [36]. The obtained K_d_ values were applied to evaluate the affinity of compounds to primary STP sludge. The K_d_ values were lowest for VEN, DVEN, and CAR (Table 2) with values ranging from 21 to 72 L/kg. With log K_d_ values ≤ 2, sorption to solid matter for VEN, DVEN, and CAR is therefore defined as negligible [36]. Higher sorption behaviour is expected for SER, DSER, FLU, and CIT which have higher relative K_d_ values (Figure 3). 

### Antidepressants in treated effluent - ozonation

**Figure 4:**
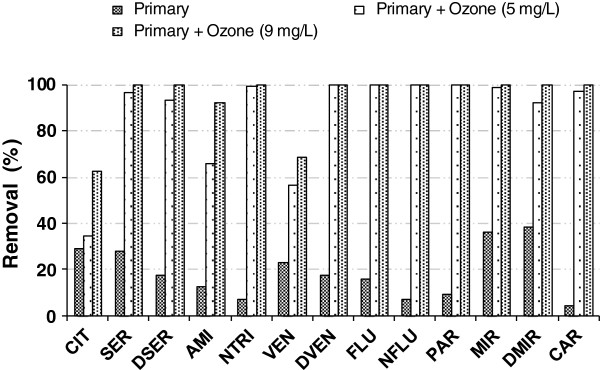
**Reduction of antidepressants and CAR in primary-treated effluent by ozone disinfection at 5 and 9 mg/L O**_**3 **_**concentration.**

Ozonation of the primary-treated effluent did degrade antidepressants with higher efficiency, yielding a mean removal efficiency of 88% when 5 mg/L of ozone was applied (Table 3). Ten (10) of the 13 compounds initially present in the effluent had removal efficiencies ≥ 92% (Figure 4). Only three substances (CIT, AMI, and VEN) yielded lower removal efficiencies, being 34, 66, and 56% respectively. As discussed in background section, the ozonation mechanism is directly affected by the ozone stability. Thus, scavengers compounds (e.g. carbonate, bicarbonate, dissolved organic and humic acids) present in effluent may have slowed down the ozone decomposition by inhibiting the free-radical reaction chain, and consequently the formation of hydroxyl radicals OH· necessary to degrade saturated aliphatic carbon chain on molecules [28]. Since, CIT, AMI and VEN have long tertiary amine aliphatic chains on their chemical structures, steric hindrance may have prevented ozone reactions normally expected at specific sites of the molecules [37]. In present study, it is very difficult to assess the relative importance of direct ozone-mediated transformations, and thereby to draw a general conclusion about each compound and transformation during ozonation in a single matrix with varying OH· scavenging capacities, under a certain pH condition. Obviously, the work presented therein was not intended to the understanding of ozonation mechanisms. However, as reported by Zwiener and Frimmel [38], so-called radical scavengers compete with pharmaceuticals for the OH-radicals and by this decrease the degradation kinetics of the targeted pharmaceuticals. Nevertheless, removal efficiency increased to 94% for most compounds using an optimal ozone dose of 9 mg/L (Figure 4). At the highest ozone treatment tested (i.e. 13 mg/L), all antidepressants were oxidized and degraded from primary-treated effluent samples. Current limitation of the analytical method may have lead to undetected polar compounds that would require different chromatographic and instrumental adjustments. However, Snyder et al. [26] have reported very similar removal efficiencies for CAR (> 99%) and FLU (> 93%) for comparable effluent samples treated with 3.6 mg/L of O_3_. Under controlled conditions using a 5–L glass jacketed reactor, Rosal et al. [39] observed high removal efficiencies for CAR (98%), CIT (93%), FLU (100%), and VEN (88%) in wastewater samples exposed to 2.4 – 6.1 mg/L of O_3_ for less than 5 min. 

**Table 3:** Mean concentrations and removal of antidepressants contained in final effluent following ozonation

**Compounds**	**Conc. (n=2) Ozone 5 mg/L**			**Conc. (n=2) Ozone 9 mg/L**		
	**Effluent (ng/L)**	**Disinfected effluent (ng/L)**	**Removal Eff. (%)**	**Effluent (ng/L)**	**Disinfected effluent (ng/L)**	**Removal Eff. (%)**
CIT	186 ± 27	123 ± 11	34	148 ± 16	56 ± 1	62
SER	14 ± 2	–	100	9.4 ± 0.1	–	100
DSER	23 ± 1	–	100	19 ± 3	–	100
AMI	106 ± 5	36 ± 1	66	195 ± 11	15 ± 1	92
NTRI	18 ± 1	0.18 ± 0.01	99	19 ± 4	–	100
VEN	2194 ± 191	963 ± 43	56	3144 ± 107	986 ± 27	69
DVEN	2319 ± 11	–	100	3448 ± 279	–	100
CAR	716 ± 4	12 ± 1	98	714 ± 13	–	100
FLU	6.3 ± 0.8	–	100	9.5 ± 0.6	–	100
NFLU	11 ± 2	–	100	6.5 ± 0.2	–	100
PAR	9.0 ± 1.3	–	100	13 ± 4	–	100
MIR	104 ± 1	1.6 ± 0.1	98	109 ± 3	–	100
DMIR	41 ± 4.1	3.3 ± 0.4	92	25 ± 1	–	100

Note: Measured residual O_3_ concentrations for 5, 9 and 13 mg/L of O_3_ were respectively 0.000, 0.036 and 0.514 mg/L.

### Characterization of new by-products by LC-QqToFMS

In this study, the two most abundant antidepressants detected in raw sewage were VEN and its *N*-desmethyl metabolite DVEN. Therefore, primary-treated effluent samples previously treated with O_3_ at different concentrations were screened by LC-QqToFMS to confirm the presence of related by-products of these two compounds.

**Figure 5:**
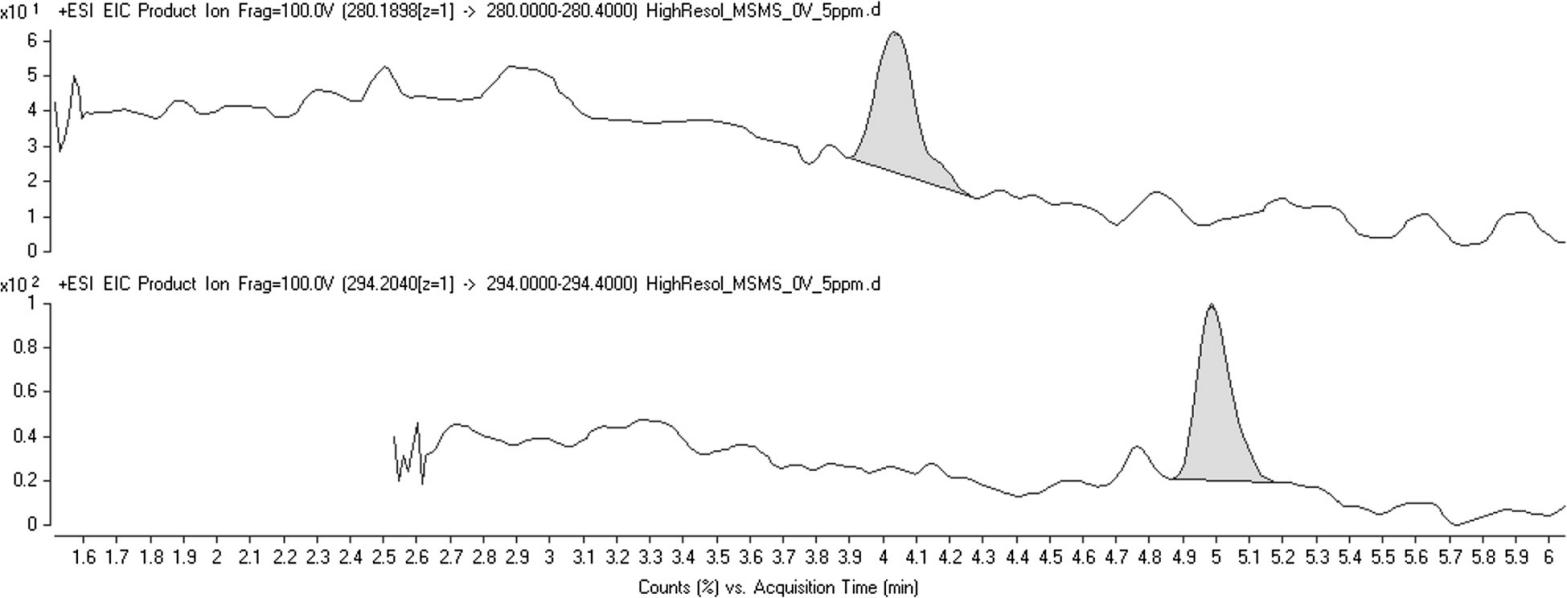
**LC-QqToFMS chromatograms of *N*-oxide by-products detected in disinfected effluent (O**_**3 **_**concentration: 5 mg/L).**

**Figure 6:**
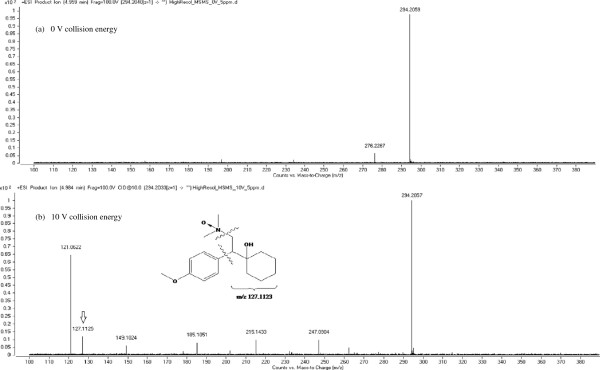
**LC-QqToFMS mass spectra of *N*-oxide VEN in disinfected effluent (O**_**3 **_**concentration: 5 mg/L): product ions at 0 V collision energy (a) and 10 V collision energy (b).**

**Figure 7:**
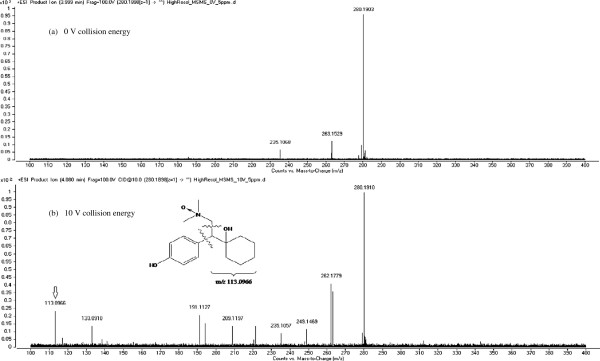
**LC-QqToFMS mass spectra of *N*-oxide DVEN in disinfected effluent (O**_**3 **_**concentration: 5 mg/L): product ions at 0 V collision energy (a) and 10 V collision energy (b).**

Initial tests performed on treated effluent extracts (O_3_ dose: 5 mg/L) in high resolution tandem MS mode using a mass range of *m/z* 100–400 (specific collision energy: 0 V) enabled the positive detection of *N*-oxide by-product precursor ions for VEN (*m/z* 294.2059, accurate mass error: -3.40 ppm) and DVEN (*m/z* 280.1903, accurate mass error: -3.21 ppm). The chromatograms and mass spectrums of both characterized by-products are depicted respectively in Figures 5, 6a, and 7a. Precursor [M + H]^+^ ions were isolated in the first quadrupole of the QqToF and then fragmented in the collision cell at 10 V in order to perform accurate mass measurements on the resulting fragment ions. Isolation and fragmentation of the precursor ion of *N*-oxide VEN (*m/z* 294.2057, accurate mass error: -4.08 ppm) generated a product ion at *m/z* 127.1125 (Figure 6b). This ion fragment corresponds to [C_8_H_14_O + H]^+^ and has an accurate mass error from theoretical values of 1.57 ppm. As for the *N*-oxide DVEN when its precursor ion at *m/z* 280.1910 (accurate mass error: –0.71 ppm) was isolated and fragmented, an ion at *m/z* 113.0966 was observed that could be interpreted as [C_7_H_12_O + H]^+^ with an accurate mass error of ± 0.00 ppm (Figure 7b). During MS/MS characterization, it was decided to keep a large isolation width of the quadrupole (e.g. 4 m/z) to increase sensitivity. Hence, MS/MS mass spectra of *N*-oxide VEN and DVEN likely contained product ions of other molecules that may have interfered with the mass spectra interpretation. According to European Commission Decision 2002/657/EC [40], at least 4 “identification” points are required in order to confirm the presence of a substance. Since one high-resolution precursor ion and one high-resolution product ion were obtained during experiments (total identification points: 2 + 2.5 = 4.5), the results of our study (with accurate mass errors < ± 5.00 ppm) were considered sufficient to confirm the presence of the *N*-oxide by-products. 

Additional LC-QqToFMS analysis performed on effluent extracts previously treated with 9 mg/L of O_3_ confirmed also the presence of both *N*-oxide by-products. When the concentration of O_3_ reached 13 mg/L, none of the by-products were detected in corresponding effluent samples. This suggests that an optimal O_3_ dosage would be required to completely degrade the *N*-oxide by-products from treated effluents. Additional tests performed on raw sewage (influent) and primary-treated effluent confirmed the absence of the two *N*-oxide by-products prior ozone treatments. To our knowledge, the present study is the first one to report the characterization of antidepressant by-products in municipal effluent samples after experimental ozone treatment.

## Conclusions

This study described the fate and behavior of antidepressants and their *N*-desmethyl metabolites in a primary STP following ozone treatment. Effluent ozonation led to higher mean removal efficiencies than current primary treatment, and therefore has represented a promising strategy for the elimination of antidepressants in urban wastewaters. However, the use of O_3_ has produced *N*-oxide by-products with unknown toxicity. Of particular concern is the potential that removal of pharmaceuticals following wastewater disinfection using advanced oxidation process (i.e. ozonation) could generate by-products of similar parent chemical structures that would need to be identified, quantified and evaluated for their toxicity.

## Competing interests

The authors declare that they have no competing interests.

## Authors’ contributions

AL performed the main part of the experiments and drafted the manuscript. MB performed ozone treatment experiments and helped analyzing the data. CG and SS helped interpreting the results and coordinated the manuscript writing. BB helped analyzing the data and drafting the manuscript. All the authors read and approved the final manuscript.

## Supplementary Material

Additional file 1 **Optimized LC-(ESI+) QqQ conditions for the analysis of antidepressants.** The supporting document reports the instrumental LC-MS/MS parameters. Click here for file
