# Supplementary material for: Ozone oxidation of antidepressants in wastewater –Treatment evaluation and characterization of new by-products by LC-QToFMS
Source: Chem Cent J. 2013 Jan 25;7:15. doi: 10.1186/1752-153X-7-15 (PMC3564768; doi:10.1186/1752-153X-7-15)
Supplement: Additional file 1 — Optimized LC-(ESI+) QqQ conditions for the analysis of antidepressants. The supporting document reports the instrumental LC-MS/MS parameters. [file 1752-153X-7-15-S1.doc]

Additional file 1. Optimized LC-(ESI+) QqQ conditions for the analysis of antidepressants.^20^

| Compounds | *t*_R_  (min) | SRM transitions  (m/z) | Fragmentor  (V) | Collision Energy  (V) |
| --- | --- | --- | --- | --- |
| Carbamazepine (CAR) | 6.62 | 237.1 → 194.0, 165.0 | 110 | 17, 50 |
| 10,11-Dihydrocarbamazepine (DHC) | 6.69 | 239.3 → 194.0 | 120 | 21 |
| Desmethylvenlafaxine (DVEN) | 6.98 | 264.2 → 246.1, 107.1 | 105 | 9, 41 |
| Desmethylmirtazepine (DMIR) | 7.89 | 252.3 → 195.1, 209.1 | 110 | 21, 21 |
| Cis-Tramadol^13^-*d*_3_ (TRA) | 8.03 | 268.1 → 250.2 | 80 | 8 |
| Mirtazepine (MIR) | 8.09 | 266.4 → 195.0, 209.0 | 115 | 21 |
| Bupropion-d_9_ (BUP) | 8.42 | 249.2 → 185.0 | 95 | 9 |
| Desmethylfluvoxamine (DFLUVO) | 8.55 | 305.3 → 229.0, 173.0 | 90 | 5, 50 |
| Venlafaxine (VEN) | 9.05 | 278.2 → 260.1, 121.1 | 95 | 9, 29 |
| Fluvoxamine (FLUVO) | 9.47 | 319.3 → 200.0, 226.0 | 95 | 21, 9 |
| Citalopram (CIT) | 9.51 | 325.2 → 109.1, 262.1 | 115 | 25, 17 |
| Desmethylsertraline (DSER) | 10.16 | 275.1 → 158.9, 123.0 | 95 | 21, 50 |
| Paroxetine (PAR) | 10.25 | 330.2 → 192.0, 135.0 | 125 | 21, 45 |
| Norfluoxetine (NFLU) | 10.78 | 296.1 → 134.2 | 75 | 5 |
| Sertraline (SER) | 11.44 | 306.1 → 158.9, 123.0 | 80 | 29, 50 |
| Fluoxetine (FLU) | 12.18 | 310.1 → 148.1 | 90 | 5 |
| Amitriptyline (AMI) | 12.27 | 278.2 → 105.1, 117.1 | 115 | 21, 21 |
| Nortriptyline (NTRI) | 13.24 | 264.2 → 105.1, 233.1 | 100 | 21, 13 |
